# Impact of F1Fo-ATP-synthase dimer assembly factors on mitochondrial function and organismic aging

**DOI:** 10.15698/mic2018.04.625

**Published:** 2018-01-30

**Authors:** Nadia G Rampello, Maria Stenger, Benedikt Westermann, Heinz D Osiewacz

**Affiliations:** 1 Department of Biosciences, Molecular Developmental Biology, Institute of Molecular Biosciences and Cluster of Excellence Frankfurt Macromolecular Complexes, J. W. Goethe University, 60438 Frankfurt, Germany.; 2 Cell Biology and Electron Microscopy, University of Bayreuth, 95440 Bayreuth, Germany.

**Keywords:** aging, F1Fo-ATP-synthase, membranes, mitochondria, remodeling

## Abstract

In aerobic organisms, mitochondrial F_1_F_o_-ATP-synthase is the major site of ATP production. Beside this fundamental role, the protein complex is involved in shaping and maintenance of cristae. Previous electron microscopic studies identified the dissociation of F_1_F_o_-ATP-synthase dimers and oligomers during organismic aging correlating with a massive remodeling of the mitochondrial inner membrane. Here we report results aimed to experimentally proof this impact and to obtain further insights into the control of these processes. We focused on the role of the two dimer assembly factors PaATPE and PaATPG of the aging model *Podospora anserina*. Ablation of either protein strongly affects mitochondrial function and leads to an accumulation of senescence markers demonstrating that the inhibition of dimer formation negatively influences vital functions and accelerates organismic aging. Our data validate a model that links mitochondrial membrane remodeling to aging and identify specific molecular components triggering this process.

## INTRODUCTION

Mitochondria are eukaryotic organelles involved in a number of essential processes including the synthesis of iron/sulfur clusters, biosynthesis of lipids and amino acids, energy transduction and generation of ATP. In aerobic organisms, most of this universal cellular energy carrier is generated by oxidative phosphorylation (OXPHOS) at the respiratory chain. The protein complexes and supercomplexes of the respiratory chain are located in invaginations of the inner membrane (IM), the so-called cristae, which occur in different shapes (e.g. tubular, lamellar) 1,2,3,4. At their basis, the cristae junctions (CJ), cristae are connected to the outer membrane (OM), which surrounds the organelle.

F_1_F_o_-ATP-synthase is an IM-bound protein complex that is essential for the electrochemical-driven generation of ATP. The individual proteins of the complex are encoded by the nuclear and by mitochondrial DNA (mtDNA). Mutations in the corresponding genes lead to F_1_F_o_-ATP-synthase deficiency, mitochondrial dysfunction 5 and severe neurodegenerative pathologies like Alzheimer’s and Parkinson’s disease 6,7,8 and have a strong impact on biological aging 9,10,11. Beside its enzymatic role, the F_1_F_o_-ATP-synthase complex plays a key role in shaping cristae structure 12,13,14. In particular, rows of F_1_F_o_-ATP-synthase dimers at the tips of the cristae appear to be critical for maintaining their typical convex curvature 15,16. Recent studies identified the impact of another protein complex, the mitochondrial contact site and cristae organizing system (MICOS) 17, in controlling IM ultrastructure 3,18,19,20. This complex is localized at the CJ. Despite the distant localization of the two complexes, interactions between them, or at least of components of them, were demonstrated. It was found that Mic10, a core component of MICOS, interacts with the dimeric F_1_F_o_-ATP-synthase and stabilizes the complex 21,22. The mechanisms regulating these interactions are largely unknown but are of key relevance for the remodeling of mitochondrial ultrastructure that is observed as adaptive response to physiological changes 23,24.

Pronounced changes of the mitochondrial ultrastructure were previously demonstrated to occur during aging of the aging model organism *Podospora anserina*. Mitochondria from juvenile cultures predominantly contain well-structured lamellar cristae with rows of F_1_F_o_-ATP-synthase dimers at their tips. During aging, the invaginations of the IM recede and the membrane curvature becomes concave forming a system of vesicles in the matrix space. This process of membrane reorganization goes along with a dissociation of F_1_F_o_-ATP-synthase dimers. Occasionally, vesicles in the matrix of old mitochondria are found in close contact with the OM at contact sites. It was suggested that at late age the OM bursts at these sites and releases mitochondrial content to the cytosol inducing programmed cell death (PCD) 25. The relevance of this series of cellular events observed during *P. anserina* aging is supported by genetic manipulations of the molecular machinery involved in the control of PCD, the final phase in the life cycle of *P. anserina *26,27. Strikingly, overexpression of a gene coding for the mitochondrial peptidyl prolyl-cis,trans-isomerase, cyclophilin D (CYPD), which is involved in the control of the mitochondrial permeability transition pore (mPTP) opening 28, leads to accelerated aging. In this overexpression strain mitochondria of young chronological age (6-days) display a vesicular ultrastructure that is characteristic for old wild-type mitochondria of 18-days of age 29. The molecular components involved in this process and its regulation were unknown.

In the yeast *Saccharomyces cerevisiae*, it was shown that dimer and oligomer formation is mediated by the interaction of two dimer assembly factors, subunit e (Atp21, Su e) and subunit g (Atp20, Su g) of F_1_F_o_-ATP-synthase 30,31. Deletion of a gene coding for either one of these proteins leads to a loss of dimers and oligomers without influencing the catalytic ATP-synthase activity of the enzyme but inducing a strong alteration of mitochondrial ultrastructure 30,32. Surprisingly, the downregulation of the gene coding for subunit *e* or *g* in HeLa cells affects not only the stability of dimers and oligomers but also the catalytic activity of the enzyme and mitochondrial morphology 33.

Here we report novel data from a study aimed to generate mechanistic insights into the impact of mitochondrial ultrastructure on biological aging. We focused on *PaAtpe* and* PaAtpg* encoding the two putative F_1_F_o_-ATP-synthase dimer assembly factors of *P. anserina* and investigated their age-related expression. Moreover, we analyzed the impact of the ablation of the two dimer assembly factors on biological aging in detail. Our data extend a model describing the age-related mitochondrial membrane remodeling including the role of F_1_F_o_-ATP-synthase dimers and dimer assembly factors PaATPE and PaATPG.

## RESULTS AND DISCUSSION

### The abundance of F_1_F_o_-ATP-synthase dimers decreases during aging 

**Figure 1:**
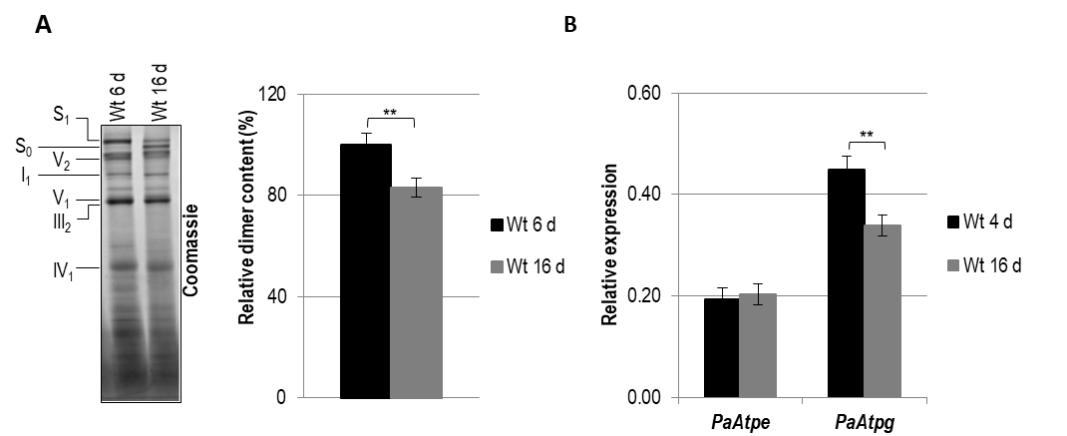
FIGURE 1: Age-dependent dimer decrease and analysis of expression of *PaAtpe* and *PaAtpg* in *P. anserina*. **(A) **Representative BN-PAGE analysis and quantification of mitochondrial protein extracts (V2, F_1_F_o_-ATP-synthase dimers) from three independent 6-days and 16-days old wild-type strains. F_1_F_o_-ATP-synthase dimers were normalized to Coomassie stained gel. Relative dimer content of 6-days old wild-type strains was set to 100%. Mitochondrial protein complexes were stained with Coomassie Blue and complexes and supercomplexes S_1_, S_0_, V_2_, I_1_, V_1_, III_1_ and IV_1 _are indicated. **(B) **Transcript analysis of *PaAtpe* and *PaAtpg* in three young (4 d) and old (16 d) wild type cultures, respectively. RNA was isolated and gene expression was determined by qRT-PCR analyses. The relative expression was normalized to the expression of *PaPorin *coding for a mitochondrial outer membrane protein. Error bars represent the standard deviation and the P-values were determined by Student´s t test.

Previous work revealed an essential role of F_1_F_o_-ATP-synthase dimers in the formation of mitochondrial cristae 12,13,14,16 and a decrease of mitochondria with lamellar cristae accompanied by an increase of those with a vesicular ultrastructure during aging of *P. anserina* 25,29. To elucidate the biochemical basis of these microscopic observations, we analyzed mitochondria from batches isolated from 6-days and 16-days old cultures of the *P. anserina* wild type that previously were used for cryo-electron microscopy 25 and subjected them to BN-PAGE analysis (Fig. 1A). We observed a 20% decrease of F_1_F_o_-ATP-synthase dimers in 16-days old cultures compared to 6-days old cultures. This effect is likely to be underestimated because of the elimination of impaired mitochondria during aging by autophagy, a quality control pathway that increases during aging 34,35. Apart from a decrease in abundance of F_1_F_o_-ATP-synthase dimers, a marked change of supercomplex amount S_1_ (I_1_III_2_IV_1_) and S_0_ (I_1_III_2_IV_0_) is observed during aging (Fig. 1A). In cells of 6-days old cultures S_1_ predominates while this supercomplex is decreased in 16-days cultures. In contrast, an increase of S_0_ is observed during aging (Fig. 1A).

Next, we investigated the age-associated expression of *PaAtpe* and *PaAtpg,* coding for two potential factors involved in the control of F_1_F_o_-ATP-synthase dimer formation. A comparative transcript analysis of these genes of young (4-days) and senescent (16-days) *P. anserina* cultures revealed a reduction of the *PaAtpg *transcript level but no significant alteration of *PaAtpe *transcripts (Fig. 1B), suggesting that the age-related regulation or stability of the two putative dimer-specific genes differs. Interestingly, in the young wild type, transcript levels of *PaAtpe *are considerably lower than those of *PaAtpg*. These observations let us speculate about different regulatory mechanisms and probably different functions of the two dimer assembly factors. It appears that PaATPE is a limiting but central factor in dimer formation. This idea is supported by different studies with yeast demonstrating that dimer assembly factors bind to the F_o_ part of ATP-synthase in a sequential manner. First Su e binds to the F_o_ part followed by the association of Su g 31,36. It was shown that the presence of Su e is required for stabilization of Su g whereas Su g is not necessary for binding of Su e 37. Thus, Su e appears to play a key role in ATP-synthase dimer formation while Su g acts as a protein supporting Su e in this function 30,31.

Overall, the observed changes in F_1_F_o_-ATP-synthase dimer quantity and alterations of supercomplexes S_1 _to S_0_ indicate mitochondrial membrane reorganization processes during aging of *P. anserina. *These biochemical data are in concordance with the observations of earlier cryo-electron microscopy studies 25,29.

### Ablation of PaATPE and PaATPG affects dimer formation and mitochondrial ultrastructure 

Next, we focused on the role of PaATPE and PaATPG in the formation of F_1_F_o_-ATP-synthase dimers. In yeast and HeLa cells the lack of one of the homologues is sufficient to inhibit F_1_F_o_-ATP-synthase dimer formation 30,31,33. In both model organisms, the deletion of either gene strongly affects mitochondrial ultrastructure. In yeast, onion-like structures of the IM are found 12. In HeLa cells, the IM forms arch-like or longitudinal cristae 33.

**Figure 2:**
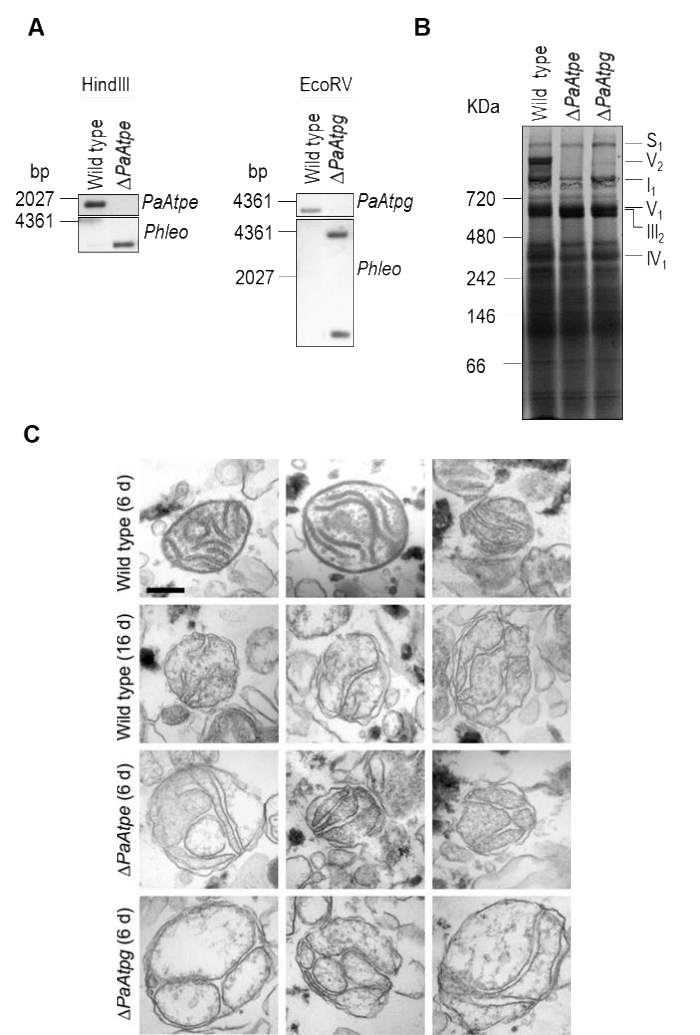
FIGURE 2: Deletion of *PaAtpe* or *PaAtpg* affects F_1_F_o_-ATP-synthase dimer formation and mitochondrial inner membrane ultrastructure. **(A) **Southern blot analysis of HindIII- and EcoRV-digested genomic DNA (gDNA) of wild-type, *ΔPaAtpe* and *ΔPaAtpg* strains. The digested gDNA was hybridized with a specific *PaAtpe*, *PaAtpg* and *Phleo* probe, respectively, and demonstrated a successful deletion of *PaAtpe and PaAtpg*. **(B)** Representative BN-PAGE analysis of mitochondrial protein extracts from 6-days old wild-type, *ΔPaAtpe* and *ΔPaAtpg* strains. Mitochondrial complexes including S_1_, V_2_, I_1_, V_1_, III_1_ and IV_1_ were visualized by Coomassie staining. **(C)** EM of isolated mitochondria of wild-type and deletion strains of different age (6 days and 16 days old). The morphology of at least 50 organelles per strain and growth condition was analyzed. Representative images are shown at the same magnification. Scale bar: 0,2 µm.

We generated *P. anserina* strains in which either *PaAtpe *(*P. anserina* accession number: Pa_1_3740) or *PaAtpg* (*P. anserina* accession number: Pa_1_2480) were replaced by a bifunctional selection marker that mediates phleomycin and blasticidin resistance 38. Strains were verified by Southern blot analysis using a specific *Phleo* and *PaAtpe *or *PaAtpg *probe (Fig. 2A). Subsequent analysis of mitochondrial extracts from each of the two deletion strains by BN-PAGE revealed the absence of F_1_F_o_-ATP-synthase dimers (Fig. 2B) and demonstrated that the *P. anserina* proteins encoded by these two genes are indeed involved in the dimerization of F_1_F_o_-ATP-synthase monomers.

Subsequently, we analyzed whether the lack of F_1_F_o_-ATP-synthase dimers affects mitochondrial ultrastructure. Electron microscopy of mitochondria isolated from young (6-days) and old (16-days) wild-type strains revealed pronounced remodeling of the IM during aging (Fig. 2C), corresponding to what was described earlier in a cryo-electron microscopy study 29. While well-separated lamellar cristae with a typical convex curvature at the tips could be readily found in preparations of young wild type mitochondria, mitochondria from old cultures were generally devoid of lamellar cristae and the inner membrane frequently formed septa and vesicular structures (reticulate ultrastructure). Mitochondria isolated from *PaAtpe* and *PaAtpg* deletion strains of young chronological age (i.e. 6-days) strikingly resemble old wild type mitochondria (16-days). Importantly, typical lamellar cristae could not be found in mutant or old wild type mitochondria (Fig. 2C). These results clearly demonstrate a direct impact of F_1_F_o_-ATP-synthase dimers on the formation of lamellar cristae and aging-dependent mitochondrial membrane reorganization.

### Defects in F_1_F_o_-ATP-synthase dimerization affects mitochondrial function and leads to accelerated aging

In a series of subsequent experiments, we concentrated on the impact of F_1_F_o_-ATP-synthase dimerization on aging of *P. anserina*. We compared well-known senescent markers in the two deletion strains and the wild type of defined chronological age. Apart from the recently identified ultrastructural reorganization of the IM, changes in mitochondrial morphotype 39, reorganization of the mitochondrial DNA (mtDNA) 40,41,42,43,44, an increase in hydroxide peroxide (H_2_O_2_) release from cultures 39,45 and impairments in respiration 46 were analyzed as hallmarks of aging.

**Figure 3:**
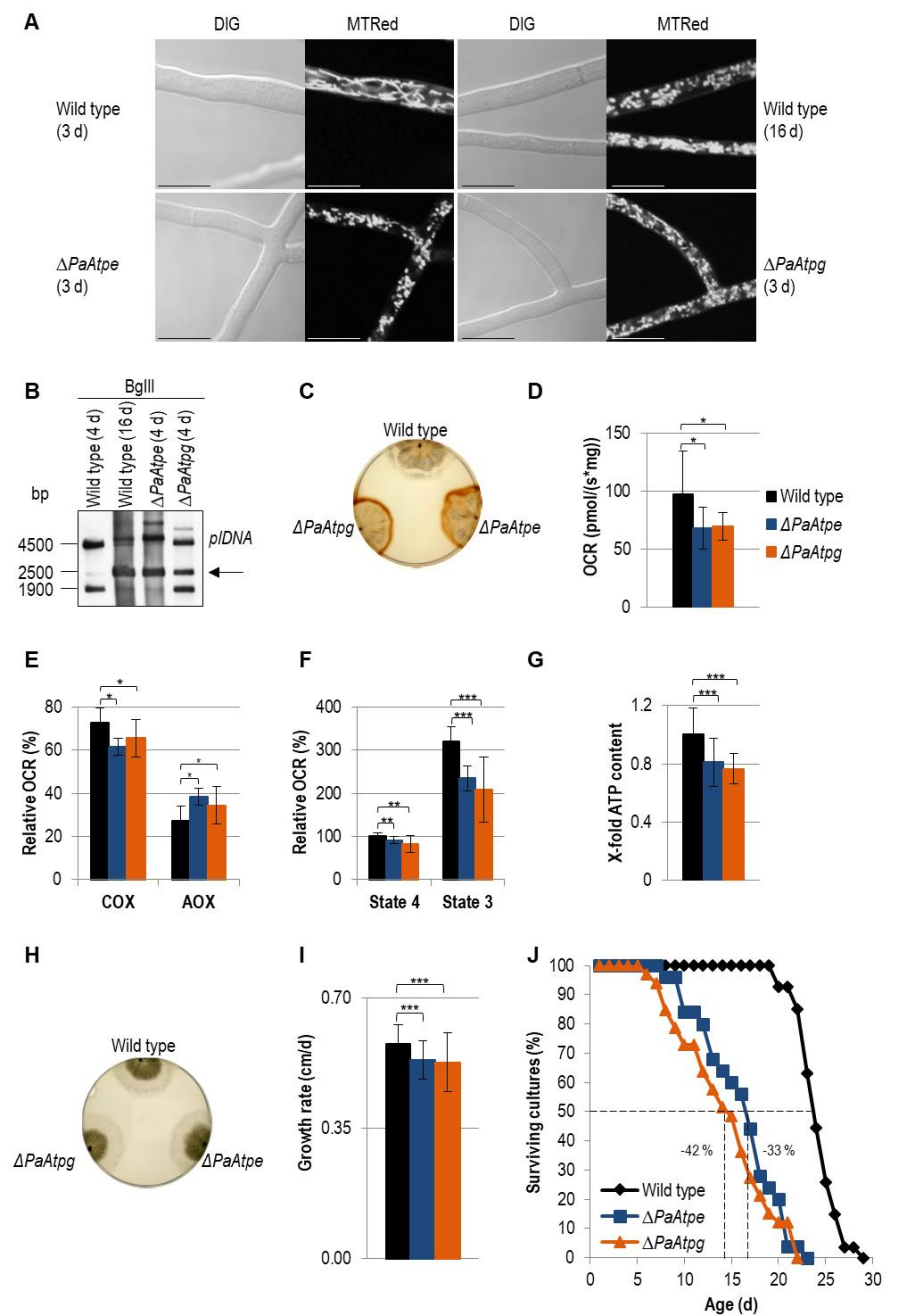
FIGURE 3: Impairments of mitochondrial functions affect growth and lifespan in deletion mutants. **(A) **Comparison of mitochondrial morphology of 3-days and 16-days old wild-type strains with those of *ΔPaAtpe* and *ΔPaAtpg *(3 d) by fluorescence microscopy. Mitochondria were stained with MitoTracker® Red and analyzed by confocal fluorescence microscopy. Scale bars: 10 µm. **(B)** Representative Southern blot analysis of BglII-digested gDNA of wild-type (4-days and 16-days old), *ΔPaAtpe* and *ΔPaAtpg *strains (4-days old). The 2500 bp age-specific *plDNA* (arrow) fragment was detected with a specific *plDNA* probe. **(C) **Release of ROS species H_2_O_2_ from wild-type (6-days n=3), *ΔPaAtpe* (6-days n=3) and *ΔPaAtpg *strains (6-days n = 3) visualized by DAB precipitation. **(D)** Oxygen consumption (basal respiration) of 6-days old deletion mutant mycelium compared to respiration of 6-days old wild type mycelium (n = 3-4 biological replicates with a total number of 12-16 technical replicates). **(E) **Relative COX- and AOX-dependent oxygen consumption rate (OCR) of 6-days old wild-type and 6-days old deletion strains mycelium after treatment with KCN (COX-inhibitor) or SHAM (AOX-inhibitor). **(F)** Relative complex I-dependent OCR of 6-days old wild-type mitochondria compared to mitochondria from 6-days old deletion mutants (n = 4-8 biological replicates with a total number of 15-28 technical replicates). State 4 OCR of mitochondria from wild type was set to 100%. **(G)** ATP production of 6-days old wild-type and 6-days old deletion mutant mitochondria after removal of samples during state 3 respiration measurements. Samples of three biological replicates and two technical replicates, respectively, were measured three times by a luminescence-based assay. **(H)** Phenotypic analysis of indicated strains growing on M2 medium for 4 days. **(I) **Growth rate of wild type (0.58 ± 0.05 cm/d, n = 27), *ΔPaAtpe* (0.53 ± 0.05 cm/d, n = 25) and *ΔPaAtpg* (0.53 ± 0.08 cm/d, n =3 3) growing on M2 medium. Growth rate was determined from day 3 to 6 of growth. **(J)** Lifespan analysis of wild type (mean lifespan = 23.3 d, n = 27), *ΔPaATPe* (mean lifespan = 15.1 d, n = 25,) and *ΔPaATPg* (mean lifespan = 13.5 d, n = 33) growing on M2 medium. The error bars represent the standard deviation and the P-values were determined by Student´s t test.

Light microscopy analysis identified an accelerated change of mitochondrial morphotypes in the two investigated deletion strains. In contrast to the wild type, in which mitochondria from 3-days old cultures are mainly of a filamentous morphotype, mitochondria of the same chronological age of the two deletion mutants display a punctate morphotype that is characteristic for mitochondria of old (i.e. 16-days old) wild-type strains (Fig. 3A).

Analysis of mtDNA revealed an accelerated amplification of a mitochondrial plasmid-like DNA (plDNA). In 4-days old *PaAtpe* and *PaAtpg* deletion strains this senescent marker is already strongly amplified. In the wild type, plDNA is almost absent from 4-days old cultures and found in strains of much older age (e.g. 16-days) (Fig. 3B).

Next, we analyzed H_2_O_2_ release 39. Incubation of wild-type and deletion strains of the same chronological age (6-days) with a diaminobenzidin (DAB) staining solution revealed an increase of a brown precipitate demonstrating a strong increase in H_2_O_2 _release by *ΔPaAtpe* and *ΔPaAtpg* cultures (Fig. 3C).

In order to link these different age-dependent changes to mitochondrial functions, we measured respiration of *P. anserina* deletion strains. Clearly, total oxygen consumption of deletion strains is reduced in comparison to the wild type (Fig. 3D). Furthermore, we determined respiration of wild-type, *ΔPaAtpe* and *ΔPaAtpg* strains in the presence and absence of respiration inhibitors potassium cyanide (KCN), inhibiting complex IV (COX), and salicylhydroxamic acid (SHAM), inhibiting the alternative oxidase AOX (Fig. 3E). Notably, in the two deletion strains alternative respiration is slightly increased as compensatory response to OXPHOS impairments and increased mitochondrial stress 46,47,48. These data are in concordance with studies in yeast in which mutations of several subunits of F_1_F_o_-ATP-synthase like Atp4 49 or Su e and Su g 50,51 lead to a destabilization of of the complex and a reduced COX activity.

Determination of the relative oxygen consumption rate (OCR) via complex I of isolated mitochondria from wild-type, *ΔPaAtpe* and *ΔPaAtpg* strains revealed that state 4 (ADP-independent) as well as state 3 (ADP-dependent) respiration is significantly decreased in deletion mutants. State 3 respiration is most strongly affected (Fig. 3F). An impaired respiration often results in an inefficient ATP production. To test F_1_F_o_-ATP-synthase activity, we removed part of the samples used for state 3 respiration measurements and determined ATP production in this state using a luciferin-luciferase assay. We found that ATP content in deletion strains is significantly reduced compared to the wild type (Fig. 3G) indicating that monomeric F_1_F_o_-ATP-synthase is not as efficient as the dimeric form. Thus, the dimerization of F_1_F_o_-ATP-synthase is not only important for F_1_F_o_-ATP-synthase dimerization and mitochondrial ultrastructure but also for energy transduction. Similar effects were also observed in yeast and HeLa cells. In HeLa cells, downregulation of dimer-specific genes leads to an increase of doubling-times and a reduction of OXPHOS capacity 33. Yeast cells in which either one of the two dimer assembly factors is ablated display an abnormal mitochondrial morphology, a decreased growth rate on non-fermentable carbon sources and reduced respiration 12,30.

The effect of reduced ATP generation in *PaAtpe* and *PaAtpg* deletion strains is not apparent in the first days (e.g. 4 days) of growth. At this time, cultures display the same morphology and radial growth rate as the wild type (Fig. 3H). Later on (days 3 - 6), growth rate of deletion strains becomes slightly, but significantly reduced when compared to the wild type (Fig. 3I). The reduction of the growth rate in the presenescent life phase is another aging marker that together with the described changes in mitochondrial morphology and ultrastructure as well as mitochondria dysfunction, reorganization of mtDNA and the increase in ROS production suggests an acceleration of the aging process in *ΔPaAtpe* and *ΔPaAtpg* strains. This conclusion was finally verified by determination of the lifespan of the two deletion strains. In comparison to the wild type, the lifespan of the mutants is strongly reduced by 33% and 42%, respectively (Fig. 3J). Until now, such an effect on biological aging was not reported in any of the different established aging models, including yeast in which F_1_F_o_-ATP-synthase dimerization factors were extensively studied.

### Conclusions and perspectives

Here we provide novel data on the identification of individual components involved in the control of mitochondrial inner membrane remodeling and their impact on biological aging. These experimental data extend a model that was proposed from earlier cryo-electron microscopy studies 25 and from data of genetic interventions into the induction of PCD via the overexpression of a gene coding for cyclophilin D, a regulator of the mitochondrial permeability transition (mPT) 29. In this particular strain, it was demonstrated that autophagy is induced to an extent that leads to autophagic cell death 52. Significantly, CYPD is known to interact with the lateral stalk of the F_1_F_o_-ATP-synthase decreasing its activity 53. Moreover, F_1_F_o_-ATP-synthase dimers were demonstrated to be part of the mPTP 54 and that opening of the mPTP correlates with the dissociation of dimers 55. Overall, these and the data presented in our current study highlight the central role of F_1_F_o_-ATP-synthase in mitochondrial biology. However, the regulatory circuits and the mechanisms involved in the different aspects of F_1_F_o_-ATP-synthase function remain to be unraveled in detail. Concerning the role in mitochondrial ultrastructure remodeling, the identification and the characterization of components involved in this process is crucial. Apart from the role of F_1_F_o_-ATP-synthase dimers at the cristae tips, the MICOS complex has been recently identified 18,19,20. Interestingly, it was found that both complexes are necessary for cristae formation. Notwithstanding that the core MICOS protein Mic60 and dimer assembly factors Su e and Su g play antagonistic roles 14, the other core MICOS protein, Mic10, was found to interact with Su e of the F_1_F_o_-ATP-synthase 21,22. Furthermore, the demonstration of a F_1_F_o_-ATP-synthase oligomerization promoting effect of Mic27 21 also suggests an interplay of the MICOS complex with the F_1_F_o_-ATP-synthase dimer. The elucidation of regulation of these crosstalks will certainly be a key aspect of future investigations.

## MATERIALS AND METHODS

### *P. anserina* strains and cultivation

In this study wild type ‘s’ 56 and the newly generated deletion strains *ΔPaAtpe* and *ΔPaAtpg* were used. Strains were grown on standard cornmeal agar (BMM) at 27°C under constant light 57. For spore germination, standard cornmeal agar (BMM) supplemented with 60 mM ammonium acetate was used and incubated at 27°C in dark for 2 days. For all experiments strains were cultivated on M2 plates for 2 days at 27°C under constant light and transferred in CM liquid medium for 2 days at 27°C under constant light. Flasks were shaken at 160 rounds per minute (rpm). All strains used in this study were derived from monokaryotic ascospores 57.

### Cloning procedures and generation of *P. anserina* mutants

Deletion of *PaAtpe* and *PaAtpg* was performed with the method described by Hamann *et al*., 2005 38. Briefly, a small 5’-flank of genes *PaAtpe* and *PaAtpg*, respectively, was amplified with oligonucleotides ATPe_KO1 (5’-GTGGTACCCGCTGTGAGAGCTTCTTC-3’), ATPe_KO2 (5’-GCAAGCTTTTTGAGGAATCTGGGGGC-3’), ATPg_KO1 (5’-CGGGTACCGTGAAGAGCGTATGTTGG-3’) and ATPg_KO2 (5’-GCAAGCTTTTCGAACTTCGGGCACTG-3’). A small 3’-flank of the same genes was amplified with oligonucleotides ATPe_KO3 (5’- GCACTAGTACTGTCCGTCGACGAACT-3’), ATPe_KO4 (5’- AAAGCGGCCGCTCAACGATGTGACATTG-3’), ATPg_KO3 (5’- CTACTAGTTCTTAGCGCAGGGAGGTG-3’) and ATPg_KO4 (5’- CAGCGGCCGCAGGTTGCAACAGTAGTAG-3’). Recognition sites of restriction endonucleases are underlined. The 5’ fragments were digested with KpnI and HindIII and the 3’ fragments with BcuI and NotI and ligated into previously digested plasmid pKO4. The resulting plasmids were termed pAtpeKO1 and pAtpgKO1 and contain a resistance cassette with *phleomycin* and *blasticidin* marker genes for fungal and bacterial selection, respectively. The flanked resistance cassettes were digested with NotI and KpnI and used for transformation of *Escherichia coli *strain KS272 bearing the plasmid pKOBEG 58 and the cosmid 15C5 containing the *PaAtpe* locus or cosmid 13B10 containing the *PaAtpg* locus, respectively. Homologous recombination between the flanks of the resistance cassette and cosmid 15C5 and 13B10 leads to generation of ∆Atpe15C5 and ∆Atpg13B10, which contain the two resistance markers surrounded by large flanks. The cosmid was isolated and used for transformation of wild type *P. anserina*. Selection of positive *PaAtpe* and *PaAtpg* deletion strains was performed by growth on medium containing phleomycin. The deletion of *PaAtpe* and *PaAtpg *was verified by Southern blot analysis as described below.

### Southern blot analysis

Total DNA was isolated according to Lecellier and Silar, 1994 59. DNA restriction, gel electrophoresis and Southern blotting were performed according to standard protocols. For Southern blot hybridization and detection, digoxigenin-labeled hybridization probes (‘DIG DNA Labeling and Detection Kit’) were used according to the manufacturer’s instructions. The *PaAtpe*-specific hybridization probe was amplified by PCR using the oligonucleotides Pa_1_3740-1 (5’-AATGGCCTCTTCCGGAGTC-3’) and Pa_1_3740-2 (5’-CTCGAGGTCGAAGCTTGAG-3’) corresponding to 511 nucleotides of the gene *PaAtpe*. The *PaAtpg*-specific hybridization probe was amplified by PCR using the oligonucleotides Pa_1_2480-3 (5’- TCTTTCGCCCTCCTCTCAC-3’) and Pa_1_2480-4 (5’- AGACGATCTTGGCCACCTC-3’) corresponding to 418 nucleotides of the gene *PaAtpg*. As a hybridization probe specific for the phleomycin resistance gene (*Ble*), the 1293 bp BamHI-fragment of the plasmid pKO4 60 was used. As a hybridization probe specific for the *plDNA* the 2500 bp BamHI-fragment of the plasmid pSP17 61 was used.

### Growth rate and lifespan determination

To determine lifespan and growth rate of *P. anserina* strains used in this study, monokaryotic ascospores were isolated from crosses of respective strains and germinated for 2 days at 27°C in the dark on BMM supplemented with 60 mM ammonium acetate. After germination mycelia were placed on M2 medium 57 and incubated at 27°C under constant light. The lifespan is defined as the period of linear growth in days (d). The growth rate was measured as growth in centimeters per day (cm/d).

### Transcript analysis

Plates containing M2 medium covered with cellophane were inoculated with small pieces of mycelium and incubated for 2 days at 27°C under constant light. Total RNA was isolated from scraped fungal mycelium using the Nucleo Spin® RNA Plant-Kit (Macherey-Nagel). Reverse transcription of 1 mg total RNA was performed with the RevertAid™ M-MuLV reverse transcriptase (ThermoScientific) according to the manufacturer’s instruction. Real-time PCR was realized using iQ SYBR Green Supermix (BioRad) followed by the manufacturer’s protocol. For each gene, the efficiency (E) of the primer pairs was calculated based on a real-time PCR with a dilution series of cDNA according to E = 10^[-1 ⁄ Slope]^ 62. The relative expression level (normalized to the level of the porin transcript) was calculated according to the following formula: Relative expression= (E(porin)^CP(porin))/(E(target gene)^CP(target gene)) with: E = PCR efficiency of the respective primer pair and CP = crossing point for each transcript.

### Mitochondria isolation

Mitochondria of *P. anserina* cultures were isolated by differential centrifugation for measurement of mitochondrial oxygen consumption and BN-PAGE analysis 57.

### Blue-native polyacrylamide gels (BN-PAGE)

BN-PAGE was performed as previously described 63. For each sample 75-100 µg of mitochondrial protein extracts were solubilized using a digitonin/protein ratio of 3:1 (w/w). Linear gradient gels (4-13%) overlaid with 3.5% stacking gels were used for separation of solubilized mitochondria. Respiratory chain components were subsequently visualized by Coomassie blue staining and assigned as described 64.

### Mitochondrial oxygen consumption

Measurement of mitochondrial oxygen consumption was performed at 27°C using two different high-resolution respirometers (Oxygraph-2k series C and G, Oroboros Instruments, Innsbruck, Austria). 200 μg freshly prepared mitochondria were injected into 2 ml air saturated oxygen buffer (0.3 M sucrose, 10 mM KH_2_PO_4_, 5 mM MgCl_2_, 1 mM EDTA, 10 mM KCl, 0.1% BSA; pH = 7.2). To determine the complex I-dependent state 4 respiration (state 4) 20 mM pyruvate and 5 mM malate were added. Thereafter, 1.5 mM ADP was added to determine complex I-dependent state 3 respiration (state 3).

Determination of complex IV- and AOX-dependent oxygen consumption was performed using strains cultivated on M2 medium for 2 days and in CM liquid medium for 2 days as described above. Small pieces of mycelium were subsequently transferred into the respirometer and oxygen consumption was measured in liquid CM medium according to the manufacturer’s instructions. 1 mM KCN was added to inhibit respiration via complex IV. 4 mM salicylhydroxamic acid (SHAM) was added to inhibit respiration via AOX.

Data were analyzed using the manufacturer´s software DatLab 6.

### ATP measurement

For determination of ATP production samples were removed after measurement of state 3 respiration. Thereafter samples were boiled in a water bath for 15 minutes. After boiling they were centrifuged for 10 min and 14,000 rpm and transferred into new reaction tubes. The supernatant was diluted (1:30) and ATP amount was measured by using the ATP Bioluminescence Assay Kit (CLS II, Roche) adapted for use in a microtiter plate format. The assay was performed according to the manufacturer’s instructions.

### Fluorescence microscopy

*P. anserina* mycelia were grown on microscope slides with a central depression that is filled with M2 agar medium and incubated for 1-2 days at 27°C and constant light. Hyphae were stained with 1 µM of the mitochondrial specific dye Mito Tracker CMX ROS (Invitrogen) and visualized using a confocal laser scanning microscope (CLSM TCS SP5, Leica).

### Electron microscopy

Electron microscopy of chemically fixed isolated mitochondria was performed as described 65.

### Hydrogen peroxide release assay

To determine the H_2_O_2_ release mycelia were cultivated on M2 medium at 27°C in the dark for 4 days. The plates were flooded with a solution containing 100 mM Tris/HCl pH 6.9 and 2.5 mM diaminobenzidine (DAB) and incubated for 30 min in the dark and 27°C. Subsequently, the solution was poured off and the plates were incubated again for 3 h at 27°C in the dark.

### Statistical analysis

All statistical significances were calculated using the Student t-test. P-values <0.05 were considered statistically significant. P <= 0.05: *, P <= 0.01: **, P <= 0.001: ***.
